# Orofacial Manifestations of Leukemic Children on Treatment: A Descriptive Study

**DOI:** 10.5005/jp-journals-10005-1510

**Published:** 2018-06-01

**Authors:** Aparna Aggarwal, Keerthilatha M Pai

**Affiliations:** 1 Reader, Department of Oral Medicine and Radiology, Maharaja Ganga Singh Dental College and Research Centre, Sri Ganganagar Rajasthan, India; 2 Professor and Dean, Department of Oral Medicine and Radiology, Manipal College of Dental Sciences, Manipal, Karnataka, India

**Keywords:** Acute lymphocytic leukemia, Descriptive study, Leukemia and oral lesions, Oral lesions in pediatric leukemia.

## Abstract

**Aim:**

To study the prevalence of orofacial manifestations in leukemic children undergoing treatment and to correlate these manifestations with various stages of chemotherapy.

**Materials and methods:**

A prospective noninvasive study comprising 43 acute lymphocytic leukemic pediatric patients at various stages of therapy. They were examined on day of their each blood examination, and lesions were recorded on a self-designed pro forma. A total of 133 observations were recorded by a single observer. The treatment was divided into the phase of induction, consolidation, maintenance, and relapse.

**Results:**

The data were analyzed using Statistical Package for the Social Sciences (SPSS) version 10.1. There were 24 males and 19 females in this study, aged between 3 and 13 years. The common oral lesions seen were dental caries, lymphadenopathy (86.04%), pallor (65.11%), ulcers (13.95%), mucositis (16.27%), gingival enlargement, hemorrhages (20.93%), candidiasis, herpes simplex virus (HSV) infection, xerostomia (44.18%), paresthesia, and tooth mobility. Herpes simplex virus infection was seen only during induction and consolidation phases. Ulcers were seen during all phases of therapy.

**Clinical significance:**

Orofacial manifestations may be seen as the first sign of leukemia and a dentist may play a significant role in the diagnosis of the disease *per se.* This study highlights not only about commonly occurring lesions but also their variation during various phases of therapy. To the best of our knowledge, no study has such an extensive reporting of orofacial manifestations of acute lymphocytic leukemia (ALL) patients under treatment.

**How to cite this article:** Aggarwal A, Pai KM. Orofacial Manifestations of Leukemic Children on Treatment: A Descriptive Study. Int J Clin Pediatr Dent 2018;11(3):193-198.

## INTRODUCTION

Acute leukemias account for most number of childhood malignancies (80%).^1-3^ There is a high incidence of orofacial manifestations. These could be seen either as initial symptoms, or as secondary findings at the time of initial examination, or found to develop secondary to treatment instituted. The common orofacial manifestations seen are hemorrhagic tendencies, gingival enlargement, lymphadenopathy, fungal, viral and bacterial infections, ulcers, mucositis, growth retardation of the jaw bones, widening of periodontal ligament space, etc.^1-5^

Oral manifestations may be seen as the first sign of the disease and a dentist may play a major role in the diagnosis of the disease *per se.* Any unexplainable oral finding along with systemic signs should always be thoroughly further evaluated with investigations. It has been reported that oral lesions may precede the changes in the blood counts.

Oral lesions may compromise nutrition intake and lead to disseminated infections, which result in increased morbidity and mortality. Therefore, early and correct detection of these manifestations may prevent the complications.

As increasing number of leukemic patients needs dental care, this study was designed to assess the prevalence of oral lesions in Indian children undergoing treatment for ALL. Another objective of this study is to know the prevalence of these manifestations in various therapies.

## MATERIALS AND METHODS

The study population comprised 43 children, aged 3 to 13 years, on treatment for ALL in Kasturba Medical College, Manipal and Kasturba Medical College, Mangaluru, Karnataka, India. The diagnosis of ALL was based on bone marrow aspiration by lumbar puncture. These children were at various stages of standard treatment protocol, including chemotherapy and cranial radiotherapy. The exclusion criteria were uncooperative or extremely ill patients. Written consent was taken from their parents. Medical records provided information regarding the demographic data and stage of therapy. The ethical committee approval was not needed as it was a noninvasive study.

**Graph 1:**
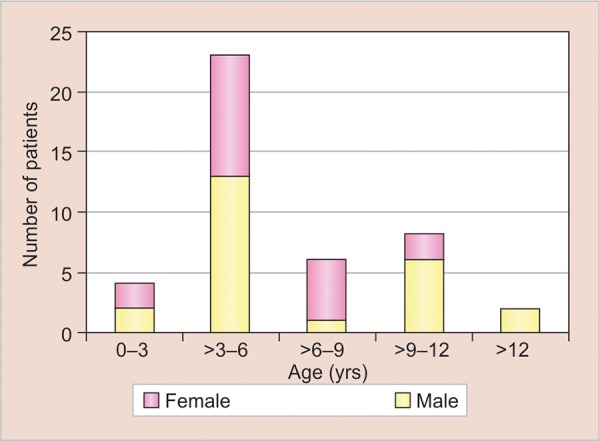
Distribution of patients according to gender and age

**Graph 2:**
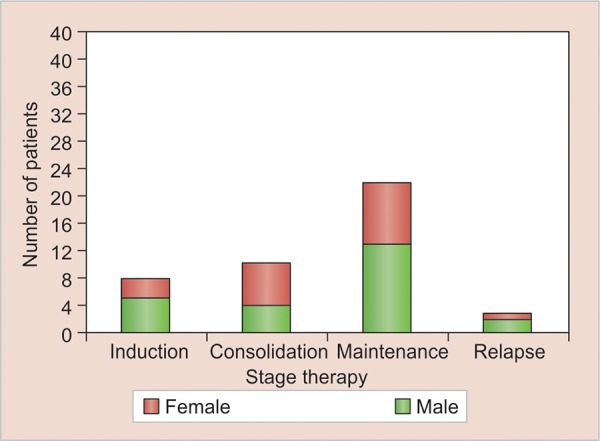
Distribution of patients according to gender and stage of therapy

The treatment was divided into the phase of induction (usually for 1 month), phase of consolidation (for 4-6 months), phase of maintenance (for 12-24 months), and phase of relapse (during any stage of treatment).

A dental specialist (Oral Medicine and Diagnosis specialist) performed extraoral and intraoral examination. Hospitalized subjects were examined on their beds, and outpatients were examined on a chair, using clean and sterile instruments, under torch light illumination. Universal precautions were followed.

Oral diagnosis was based on clinical findings. The presence of oral lesions and their locations were recorded on a proforma designed for the study. Repeated examinations were performed on all subjects on the day of their each blood examination. Each oral examination was considered an observation.

All children received prophylactic benzydamine hydrochloride mouth rinse (to relieve symptoms of mucositis), and topical 1% clotrimazole mouth paint (to prevent oral candidiasis).

Data analysis was performed using SPSS, Windows version 10.1. To calculate the prevalence of oral lesions, only the findings of first observation of each patient were used. The prevalence of the oral lesions was calculated as percentages of the total. The prevalence of oral lesions in various stages of treatment was calculated as percentages of the total observations.

## RESULTS

There were 24 males and 19 females in this study. Graph 1 shows the prevalence of ALL in various age groups according to the gender. Twenty-three (53.48%) patients were in the age group of 3 to 6 years. Most of the patients in the age group of 6 to 9 years were females.

Graph 2 shows the distribution of the subjects according to the stage of the therapy during which examinations were conducted. Most of the patients were in the maintenance phase followed by consolidation, induction, and relapse phase.

The prevalence of orofacial manifestations according to gender is presented in Table 1. Dental caries showed highest prevalence of 88.37% (38 subjects), occurring more in males (51.16%) as compared with females (37.2%), followed by lymphadenopathy and pallor. Herpes simplex virus lesions were not observed in any patient. Other common lesions seen were xerostomia, cushingoid facies, hemorrhage, mucositis, candidiasis, and ulcers.

Table 2 shows the distribution of orofacial manifestations according to the stages of therapy: Induction, consolidation, maintenance, and relapse. A total of 22 observations were made during the induction stage, 60 observations during consolidation stage, 42 observations during maintenance stage, and 9 during relapse protocol of therapy. Induction phase showed more positive observations except for cushingoid facies, candidiasis, and nerve involvement. Gingival enlargement was only seen during maintenance and relapse phases.

**Table 1:** **Table 1:** Prevalence of oral lesions according to gender

		*Male (M)*		*Female (F)*			
*Oral manifestations*		*(n = 24)*		*(n = 19)*		*Total*	
Dental caries		22 (51.16%)		16 (37.2%)		38 (88.37%)	
Lymphadenopathy		21 (48.83%)		16 (37.2%)		37 (86.04%)	
Pallor		16 (37.2%)		12 (27.9%)		28 (65.11%)	
Xerostomia		14 (32.5%)		5 (11.62%)		19 (44.18%)	
Cushingoid facies		8 (18.6%)		7 (16.27%)		15 (34.8%)	
Hemorrhage		7 (16.27%)		2 (4.65%)		9 (20.93%)	
Mucositis		5 (11.62%)		2 (4.65%)		7 (16.27%)	
Candidiasis		5 (11.62%)		1 (2.32%)		6 (13.95%)	
Ulcer		4 (9.3%)		2 (4.65%)		6 (13.95%)	
Teeth mobility		3 (6.97%)		1 (2.32%)		4 (9.3%)	
Nerve involvement		2 (4.65%)		0		2 (4.65%)	
Gingival enlargement		0		2 (4.65%)		2 (4.65%)	
HSV infection		0		0		0	
Others		4 (9.3%)		3 (6.97%)		7 (16.27%)	

**Table 2:** **Table 2:** Distribution of orofacial manifestations according to the stage of therapy

		*Induction (Total = 22)*		*Consolidation (Total = 60)*		*Maintenance (Total = 42)*		*Relapse (Total = 9)*	
Dental caries		22 (100%)		59 (98.33%)		35 (83.33%)		9 (100%)	
Pallor		22 (100%)		58 (96.67%)		27 (64.28%)		8 (88.88%)	
Lymphadenopathy		22 (100%)		36 (60.0%)		36 (85.71%)		8 (88.88%)	
Xerostomia		16 (72.72%)		47 (78.33%)		21 (50.0%)		7 (77.77%)	
Cushingoid facies		8 (36.36%)		50 (83.33%)		13 (30.95%)		7 (77.77%)	
Hemorrhage		12 (54.54%)		23(38.33%)		5 (11.90%)		1 (11.11%)	
Ulcer		13 (59.10%)		20 (33.33%)		2 (4.76%)		2 (22.22%)	
Candidiasis		5 (22.27%)		19 (31.67%)		3 (7.14%)		4 (44.44%)	
Mucositis		11 (50.00%)		13 (21.66%)		3 (7.14%)		2 (22.22%)	
Teeth mobility		8 (36.36%)		8 (13.33%)		1 (2.38%)		0 (0%)	
Nerve involvement		2 (9.09%)		8 (13.33%)		3 (7.14%)		0 (0%)	
HSV infection		4 (18.18%)		4 (6.66%)		0 (0%)		0 (0%)	
Gingival enlargement		0 (0%)		0 (0%)		3 (7.14%)		1 (11.11%)	

## DISCUSSION

Acute lymphocytic leukemia is characterized by a high incidence of wide range of orofacial manifestations, which may be the initial presenting symptoms, or a sign or secondary to treatment instituted. This study was designed to assess the prevalence of oral lesions and their distribution according to the stage of therapy.

The study population comprised 43 children undergoing treatment for the first time, or consecutively, for acute lymphoblastic leukemia. Medical records were accessed for blood counts, and oral examinations were performed by a trained oral physician on the day of these blood counts. The data were collected and the results were analyzed using SPSS version 10.1.

There were 24 males and 19 females in this study. Of these patients, 23 (53.48%) were in the age group of 3 to 6 years. This finding was similar to that of Curtis.^1^ Of these 23 patients, 13 (56.5%) were males and 10 (43.5%) were females. This finding was similar to that of Michaud et al.^2^ It was observed that as the age advanced from 9 years and above, more males were affected.

According to the stage of treatment, 8 (18.6%) subjects were in the induction phase, 10 (23.25%) in consolidation phase, 22 (51.16%) in maintenance phase, and 3 (6.97%) were on relapse protocol in the present study.

Dental caries showed high prevalence of 88.37% (38 subjects). Here again, the rate was higher in males (51.16%), as compared with females (37.2%). Fleming and Kinirons,^3^ Nunn et al,^4^ and Sonis et al^5^ could not show any significant difference in the incidence of dental caries. Cubukcu and Gunes^6^ have reported increase in the caries (53%) after initiating the therapy. As dental caries evaluation prior to developing ALL in the subjects was not done or no control group was included in this study, to say that these patients showed increased prevalence of caries would not be justified. The prevalence of dental caries among healthy Indian children has been found to be much lower by Saravanan et al^7^ (44.4%), David et al^8^ (27%), and Mahejabeen et al^9^ (54.1%). Also, other factors like reduced salivary flow, poor oral hygiene, soft diet, etc., which all could affect dental caries status of the patient were not considered in this study.

Lymphadenopathy was seen as the second most prevalent orofacial finding occurring in 37 (86.04%) subjects. The occurrence was higher in males (48.83%) as compared with females (37.2%). A much less prevalence of lymphadenopathy was reported by Michaud et al^2^ (31.7%) and Baliga et al^10^ (12.77%), whereas Barrett^11^ showed results similar to our study (75%). The most commonly involved lymph nodes group in this study was the submandibular nodes.

Pallor was the third most prevalent finding (65.11%) with occurrence of 37.2% in males. A much lower prevalence of pallor was reported by Curtis,^1^ Michaud et al,^2^ and Baliga et al.^10^

In this study, the overall prevalence of xerostomia was 44.18% (19 subjects), with a higher prevalence in males (32.5%) as compared with females (11.62%). Williams and Martin^12^ and Sonis et al^5^ found no significant changes in salivary flow in their patients.

Cushingoid facies was observed in 34.85% of the patients with an almost equal distribution among males and females. This observation is attributed to the use of corticosteroid, therefore, similar distribution pattern was observed. Michaud et al^2^ reported cushingoid facies in 20.6% of their subjects, which is lesser than our observation.

The overall prevalence of hemorrhage observed here was 20.93% seen in nine patients. Most of these were observed in males (16.27%) as compared with females (4.65%). This study showed much less prevalence of hemorrhages as compared with that of Lynch and Ship (56%),^13^ Curtis,^1^ and Michaud et al.^2^ Our findings were similar to Dreizen et al (13.1%)^14^ and Barrette (22.1%).^15^ Petechiae were observed in 6 (66.67%) patients, ecchy-mosis in 1 (11.11%), gingival bleeding in 1 (11.11%), and petechiae and gingival bleeding in another (11.11%) patient. Baliga et al^10^ reported much lower incidence of petechiae (6.4%) as an initial finding.

Mucositis was observed in 7 (16.27%) subjects, with 11.62% occurring in males and 4.65% in females. Childers et al (48.77%)^16^ and Figliolia et al (46%)^17^ reported much higher incidence of mucositis. Mucositis was graded according to World Health Organization criteria.

Candidiasis was observed in 6 (13.95%) subjects; more commonly in males (11.62%), which was similar to the study by Childers et al,^16^ but less when compared with other studies.^2,15,18^ This decline in the rate of can-didial infection could be due to better maintenance of oral hygiene and use of prophylactic antifungal agents. Angular cheilitis was observed in 2 patients, median rhomboid glossitis in another one, and pseudomembra-nous in the other 3 patients.

The overall prevalence of ulcers was 13.95%, observed in 6 subjects. These ulcers were more frequently seen in males (9.3%) than in females (4.65%). Earlier studies show a higher prevalence of 53,^19^ 36.5,^2^ 65,^20^ 33,^21^ 65,^22^ and 91.77%.^16^ Four patients showed neutropenic ulcers and remaining two had obvious cause of trauma. Sepulveda et al^23^ reported a strong association of HSV organisms with oral ulcers (in 7 out of 10 lesions).

Tooth mobility was seen only in 4 (9.3%) subjects; mostly occurring in males (6.97%). Michaud et al^2^ reported prevalence of tooth mobility to be 4.7%. This finding was seen in 4 patients, two of which showed physiologic mobility in their lower anterior teeth, while the other two were above 12 years of age and showed mobility in their permanent teeth.

Nerve involvement was noticed in only two male subjects (4.65%). One patient showed involvement of facial nerve Bell’s palsy. Other patient had facial nerve palsy along with right hypoglossal nerve palsy. In literature facial paralysis, trigeminal neuralgia, inability to protrude the tongue, difficulty in swallowing, weakness in biting, and paresthesia or anesthesia of the face, lips, or tongue have been reported to be associated with ALL.^2,24^

Gingival enlargement was observed in 2 (4.65%) females. This was in concurrence with Michaud et al,^2^ while Lynch and Ship^13^ observed a higher prevalence (36%). The HSV lesions were not observed in any patient during the first observation. Lynch and Ship (3%),^13^ Michaud et al (7.9%),^2^ and Huber and Terezhalmy^25^ showed very high incidence of secondary lesions (4070%) and primary being only <2%.

Other findings like macroglossia, fissured tongue, loss of taste sensation, burning sensation, hyperpigmen-tation of the oral mucosa, geographic tongue, and soft palate fistula were observed in 7 subjects. Michaud et al^2^ reported soft palate defects in 3.1% patients.

Out of the total 133 observations, 22 were made during the induction stage, 60 observations during consolidation stage, 42 observations during maintenance stage, and 9 during relapse protocol of therapy.

Dental caries was observed with great prevalence in all the phases—100% in induction and relapse phase, 98.33% in consolidation phase, and 83.33% in maintenance phase. It would be unscientific to consider these figures to be true. First of all, caries is a multifactorial disease, secondly neither the caries status of these patients prior to developing ALL was known nor was any control group included to compare the same. Previous studies have shown no difference in the dental health status of leukemic children and their siblings, and controls.^3,5,12,26^ Dholam et al^27^ have reported a 33% increase in dental caries during induction phase. They correlated this rise in dental caries to changes in the oral environment due to aggressive induction therapy.

Pallor was a consistent finding during almost all the stages of therapy. Pallor was observed in all subjects during induction phase and 96.67% subjects during consolidation phase of treatment. This finding suggests anemia either due to myelosuppression or mucosal bleeding mainly in the viscera. During the relapse and maintenance phase, pallor was seen in 88.88 and 64.28% observations respectively.

The prevalence of lymphadenopathy was less in consolidation phase subjects (60%), as compared with those in induction (100%), relapse protocol (88.88%), and maintenance phase (85.71%) of therapy.

Xerostomia was observed with almost equal prevalence during induction (72.72%), consolidation (78.33%), and relapse (77.77%) phases. At only 21 (50%) observations during maintenance phase reduced salivary flow was noticed. Xerostomia can develop due to chemotherapy (reversible) as well as cranial radiation (dose-dependent) given to these subjects.^2^ Our finding is in contrast with that of Williams and Martin^12^ and Sonis et al.^5^

Cushingoid facies, which is a reversible appearance related to the administration of corticosteroids, was seen in 83.33% observations during consolidation, 77.77% observations in relapse phase, 36.66 and 30.95% during induction and maintenance phase respectively. Baliga et al^10^ in their study found cushingoid facies among 93.61% of 47 subjects during the induction phase, and directly correlated the changes to the administration of drug prednisolone.

It was observed that hemorrhages were more during induction phase (54.54%), followed by consolidation phase (38.33%), maintenance phase (11.90%), and relapse phase (11.11%). These differences in the observations are probably based on the intensity of the treatment, and bone marrow invasion due to the disease *per se.* This finding was in contrast with that of Dreizen et al,^14^ whereas Wahlin and Matsson^18^ reported a similar observation (54%) during the induction phase. This variability in results may have occurred due to the short duration of the present study, wherein complete follow-up of the subjects through all the stages of treatment was not possible.

The ulcers were present at 13 (59.09%) observations during induction phase, 20 (33.33%) observations during consolidation phase, 2 (4.76%) observations during maintenance phase, and at 2 observations (22.22%) during relapse phase. Similar to our study, Wahlin and Matsson^18^ observed a higher incidence of oral ulcers (69%) during the induction phase. Baliga et al^10^ observed oral ulcers in only 47.53% of their subjects on induction therapy. Dholam et al^27^ reported of palatal aphthous ulcer in only one of their 33 patients during the induction phase.

The ulcers mainly occur due to neutropenia but the other contributing factors can be atrophied mucosa, trauma, radiation-induced mucositis, and xerosto-mia.^16,28,29^ Due to the multiple drug therapy at all the stages, to infer a direct relationship of administration of each drug (methotrexate, fluorouracil, cytarabine, etc.), oral ulcers were not possible.

Candidiasis was observed during all the phases of treatment. The prevalence was highest among the relapse phase subjects (44.44%), followed by consolidation phase (31.67%), induction phase (22.27%), and lastly, 7.14% in maintenance phase. The infection occurs despite the use of prophylactic of 1% clotrimazole solution, which suggests decreased immunity, and other contributing factors like increased use of antibiotics, xerostomia, and poor oral hygiene.^30^ Fleming and Kinirons^28^ and Baliga et al^10^ observed a higher prevalence (31 and 48.93% respectively) of candidiasis during induction phase. Dholam et al^27^ observed only one case of angular cheilitis during induction phase.

Mucositis was observed in 11 (50%) observations during induction phase, followed by 2 (22.22%) during relapse phase, 13 (21.66%) during consolidation phase, and 3 (7.14%) during maintenance phase. Baliga et al^10^ reported of similar incidence of mucositis and erythema (48.93%) in their subjects during induction phase. Pels^31^ reported of mucositis in 44.8% during induction and 3.17% during maintenance phase. She correlated the severity of mucositis directly to administration of metho-trexate. Mucositis can develop due to chemotherapy as well as cranial radiation given to these subjects.

Mobility of the teeth was observed eight (36.36%) times during induction, eight (13.33%) times during consolidation, and once (2.32%) during maintenance phase.

Nerve involvement was observed eight times (13.33%) in consolidation phase, two times (9.09%) in induction phase, and three times (7.14%) in maintenance phase. This was observed in total 5 patients, when their condition was deteriorated, and it persisted throughout the observation period. Michaud et al^2^ have reported facial nerve palsy in leukemic children. Hiraki et al^24^ reported hypoglossal nerve palsy to be quite rare.

Herpes simplex virus infection was observed in the same patient during induction (18.18%) and consolidation phase (6.66%). Baliga et al^10^ showed a lower incidence (10.63%) during induction phase. Both recurrent herpes labialis as well as intraoral herpes occurred in our patient.

Inflammatory gingival enlargement was observed once (11.11%) during relapse phase and thrice (7.14%) during maintenance phase. As leukemic infiltration is more commonly seen in acute myelocytic leukemia, biopsy was not done to confirm the same. Our finding was in consensus with that of Lynch and Ship (36%)^13^ and Michaud et al (4.7%).^2^ Curtis^1^ did not observe gingival enlargement in any of his patients.

There were few limitations in this study. All the patients could not be observed at all the stages of their treatment due to the short duration of the study. There was no previous records of dental caries of any of our patients to say that the disease ALL or its treatment had affected the caries status. Oral lesions like HSV or ulcers could not be confirmed by specific investigations due to poor health of the patients.

## CONCLUSION

This study was carried out in lieu of collection of data from India following the advancements in treatment of ALL. It is a humble effort to give a detailed overview of oral lesions in ALL patients on treatment. The data provided should help a dentist or a physician to provide a better care as well as to bring more interest in this area of research as ALL is the most common malignancy of childhood.

## CLINICAL SIGNIFICANCE

Orofacial manifestations may be seen as the first sign of leukemia and a dentist may play a significant role in the diagnosis of the disease *per se.* This study highlights not only about commonly occurring lesions but also their variation during various phases of therapy. To the best of our knowledge, no study has such an extensive reporting of orofacial manifestations of ALL patients under treatment.
